# In Their Own Words: An Innovative Approach to Intergenerational Learning in Aging Courses

**DOI:** 10.1093/geroni/igaf122.3512

**Published:** 2025-12-31

**Authors:** Carly Chak, Eleni Vodantis, Nicole Alea

**Affiliations:** University of California, Santa Barbara, Santa Barbara, California, United States; University of California, Santa Barbara, Santa Barbara, California, United States; University of California, Santa Barbara, Santa Barbara, California, United States

## Abstract

Educational initiatives that encourage younger adults to interact with older adults is one way to reduce negative age-related stereotypes in university students. Incorporating intergenerational interactions into aging-related courses can be challenging when the class size is large. To address this challenge, this project included the development of pre-recorded “Aging Insight Videos”. The videos feature five to six different older adults of varying backgrounds and lived experiences. The older adults answer instructor-developed questions that link with course concepts (e.g., social relationships, cognitive aging). These videos provide students the opportunity to learn about course material directly from the life experiences of older adults, in their own words. The current presentation focuses on: how older adult interviewees were chosen, the development of the questions, how the videos were edited, and ideas for implementing the videos into aging courses. Initial evidence, from week one of our adult development and aging course (N = 125), in which the videos were not included, suggests this instructional pedagogy is needed. Students held implicit ageist attitudes: older adult faces were rated significantly lower across five positive traits (warmth, competence, likeability, physical health, memory ability) compared to middle-aged and younger adult faces. We anticipate perceptions to positively change by the end of the course. However, the discussion will focus on how inclusion of the Aging Insight Videos as part of the course curriculum, and having students learn from and interact with older adults through these videos, in a next iteration of the course, should further reduce age-related negative stereotypes.

